# Dynamics of Nutrient Components and Microbial Communities in Substrates During the Development of the Fruiting Bodies of *Volvariella volvacea*

**DOI:** 10.3390/jof11070479

**Published:** 2025-06-24

**Authors:** Le Wang, Qin Dong, Qian Guo, Lei Zha, Lin Yang, Changxia Yu, Yan Zhao

**Affiliations:** 1 Shanghai Academy of Agricultural Sciences, Shanghai 201403, China; wangle202108@126.com (L.W.); maomao88719@163.com (Q.D.); guoqian@saas.sh.cn (Q.G.); zhalei@saas.sh.cn (L.Z.); ylin_jade@163.com (L.Y.); ycx41529@163.com (C.Y.); 2 Shanghai Key Laboratory of Agricultural Genetics and Breeding, Shanghai 201106, China

**Keywords:** circular agriculture, *Volvariella volvacea*, fruiting body development, nutrient composition, microbial community

## Abstract

Cotton waste, a growth medium for *Volvariella volvacea*, has significant commercial and nutritional value. Under controlled environmental conditions, substrate nutrient composition and microorganisms affect the growth of *V. volvacea*. In this study, the changes in the nutrient content of the substrate at different stages of fruiting body development were compared based on an 86% waste cotton substrate, and microbial diversity was studied via 16S rRNA analysis. The results indicated that there were significant differences in nutrient content in the substrate at different stages of fruiting body development. The total contents of carbon, nitrogen, and phosphorus initially increased but then decreased due to nutrient absorption and utilization by *V. volvacea*. It was also found that large amounts of organic nitrogen decomposed into more readily utilizable inorganic nitrogen. The nutritional content and microbial community structure of the substrate during the egg stage significantly differed from those during the other four stages, making the egg stage the most critical period in cultivation. Through correlation analysis between nutrient content and microbial differences, it was found that differential microbial taxa (Beijerinckiaceae, Burkholderiales, *Chitinophaga jiangningensis,* etc.) with nitrogen fixation, denitrification, and cellulose decomposition functions were significantly related to carbon- and nitrogen-related indicators such as nitrate nitrogen, microbial biomass carbon, and alkali-hydrolyzed nitrogen. These microorganisms play important roles in determining the variation in the nutritional profile of the substrate. This study provides a theoretical basis for promoting the absorption and utilization of nutrients by *V. volvacea* by altering the structure of the microbial community of the growth substrate.

## 1. Introduction

*Volvariella volvacea* (Bull.) Singer is highly valued in tropical and subtropical regions because of its rapid growth, unique flavor, and high nutritional value [1,2]; it belongs to the family Pluteaceae of the order Agaricales under division Basidiomycota [3]. Studies have shown that bioactive compounds in *V. volvacea*, such as polysaccharides and proteins, have various health benefits, including immune enhancement, cholesterol reduction, blood sugar regulation, antitumor effects, and anticancer effects [4]. In addition, *V. volvacea* contains various mineral elements, such as phosphorus, potassium, and calcium, which are indispensable elements for maintaining normal life activities in the human body [5,6]. The rich variety of amino acids also gives it high nutritional value [7]. *V. volvacea* is a high-temperature fungus and is produced in summer [8,9]. However, the majority of edible fungi currently cultivated are of medium- and low-temperature varieties, and high-temperature varieties are extremely rare; this leads to a shortage of edible fungal varieties on the market during the high-temperature season. These factors also cause the price of *V. volvacea* to remain high throughout the year, which can increase the profits of mushroom farmers compared with those of other edible fungal varieties.

In addition to its notable nutritional and economic value, the ecological benefits of *V. volvacea* cultivation merit significant attention. Commercial production of *V. volvacea* predominantly utilizes agricultural byproducts such as waste cotton, rice straw, spent mushroom substrate, and sugarcane bagasse, which serve as cost-effective and sustainable growth media [10,11,12]. The use of waste cotton as a cultivation substrate has garnered widespread interest as an environmentally sustainable waste management approach. Waste cotton is an organic residue with a large global output. This abundant material is rich in macromolecular organic compounds, including hemicellulose, cellulose, lignin, and starch, which can provide essential nutrients for *V. volvacea* growth [13,14,15,16]. The cultivation of *V. volvacea* using waste cotton is an efficient and economical biotechnology that can transform agricultural waste into high-quality food, exemplifying circular agricultural practices [17,18]. Compared with other substrates, the use of waste cotton to cultivate *V. volvacea* can achieve relatively high and stable yields while simultaneously enhancing the quality of *V. volvacea*. Consequently, it has become the predominant substrate in large-scale commercial cultivation. However, owing to the gradual increase in the utilization rate of cotton by cotton mills in recent years, the availability of waste cotton has gradually decreased, resulting in a reduction in the nutrients in waste cotton that can be utilized by *V. volvacea*. This decline has resulted in markedly inferior yields, nutritional qualities, and flavor profiles in *V. volvacea* produced with waste cotton substrates. Therefore, enhancing nutrient conversion efficiency during cultivation has emerged as a critical challenge for improving both the productivity and quality of *V. volvacea* production.

Previous research on *V. volvacea* cultivation has focused primarily on substrate formulation, environmental conditions (e.g., temperature and humidity), fermentation processes, and basic nutritional requirements during mycelial growth [11,19]. Numerous studies have focused on microbial community changes in mushroom substrates during fermentation and growth phases. For example, Souza et al. [20] investigated thermophilic fungal populations during phase II composting of *Agaricus subrufescens*, identifying *Scytalidium thermophilum* as the dominant species throughout the process. This study is the first to report the involvement of *Theromyces badanensis* in substrate fermentation for *A*. *subrufescens* production. In studies on *V. volvacea* cultivation, Chen et al. [21] documented bacterial community dynamics during phase II composting and reported the highest bacterial diversity during the high-temperature stage. The dominant microorganisms throughout fermentation were heat-resistant bacteria and fungi, with most bacteria identified as Bacteroidetes, Proteobacteria, and Firmicutes. Similar findings have been reported in the substrates of mushrooms such as *Agaricus bisporus* [22,23,24] and *Pleurotus* spp. [25].

During the growth cycle of *V. volvacea*, the vegetative growth stage begins first, and then it transitions into the reproductive growth stage, where the hyphae knot and form primordia, initiating the development of the fruiting body. The development of the fruiting body can be divided into five stages: the needle stage, the button stage, the egg stage, the elongation stage, and the maturation stage, which typically range from the 7th to the 14th day after sowing [26]. However, *V. volvacea* presents distinct nutritional requirements at different growth stages, particularly during fruiting body development, when nutrient demand increases significantly [27]. Currently, little is known about the interaction between the nutritional composition of compost and associated microbiota during this critical phase. Microbial communities in cultivation substrates are known to influence nutrient cycling (e.g., cellulose degradation, nitrogen fixation) and pathogen suppression, thereby indirectly affecting fungal growth [28,29]. For example, in *A. bisporus* cultivation, specific bacterial taxa (e.g., *Pseudomonas*) increase nitrogen availability through organic matter decomposition [22,30]. Studies on composting have revealed that Actinobacteria (particularly Actinomycetales) and Firmicutes (Bacillales and Clostridiales) play crucial roles in cellulose and lignin degradation, serving as primary decomposers of complex carbohydrates (e.g., sugarcane byproducts) [23,31,32,33]. Additionally, bacterial communities contribute to pathogen defense and promote mycelial and fruiting body growth. Cho et al. [34] isolated *Pseudomonas* spp. from *Pleurotus ostreatus* mycelia and demonstrated their positive effects on fruiting body formation and development. Proteobacteria, particularly *Pseudomonas putida*, have been shown to stimulate the formation of fruiting bodies [35]. Similar interactions may occur during *V. volvacea* fruiting body development, but systematic studies linking microbial succession with substrate nutrient dynamics during this period are lacking. Moreover, unlike other cultivated mushrooms, *V. volvacea* has an exceptionally short growth cycle (typically 10–14 days), making its nutrient uptake dynamics and interactions with the substrate microbiota particularly crucial for yield and quality optimization.

Therefore, in this study, we collected substrate samples during the fruiting body development stage of *V. volvacea* and investigated (1) changes in nutrient composition; (2) changes in microbial community structure; and (3) the relationships between microorganisms and the nutrient profile of the substrate, providing a theoretical basis for promoting the absorption and utilization of nutrients by *V. volvacea* by changing the structure of the microbial community in the substrate, promoting the efficient utilization of agricultural waste, and improving the ecological and economic benefits of *V. volvacea* cultivation.

## 2. Materials and Methods

### 2.1. Cultivation Method and Sampling

The present work was conducted at the ZhuangHang Experimental Base at the Shanghai Academy of Agricultural Sciences. The *V. volvacea* strain V23 was provided by the Shanghai Fanshun Edible Fungi Professional Cooperative. Waste cotton was selected as the culture material for *V. volvacea* cultivation. The formula used was as follows: 86% waste cotton, 8% bran, 4% cornmeal, and 2% light calcium were fully mixed according to the appropriate proportion, and the water content was adjusted to 70% [2]. Bed rack culture was adopted in the cultivation room, and the thickness of the substrate was 12 cm. The culture material was pasteurized after it reached the cultivation room and kept at a temperature of 58–62 °C for more than 8 h. After pasteurization, the temperature was cooled naturally, and the spawn was sown via the broadcasting method when the temperature was reduced to 40 °C and the sowing amount was 1%. After sowing, the air temperature of the cultivation room was controlled to 34 °C, and the air humidity was 90%.

Six days after sowing, the mycelia fully colonized the substrate, and water and light were supplemented. During this period, the air temperature was adjusted to 30–32 °C while the same air humidity was maintained, and regular ventilation was ensured. The experiment was set up with 3 replicates, and the temperature, light, and moisture conditions in each replicate were consistent. Samples from the needle stage (A), the button stage (B), the egg stage (C), the elongation stage (D), and the maturation stage (E) were collected via the five-point method at 0–5 cm below the surface of the substrate to analyze the nutrient content of the substrate and the structure of the microbial community. The whole cultivation period was 18 days.

### 2.2. Nutrient Content Analysis

The nutrient content of the substrate samples from 5 stages (A–E) was detected. The water content (WC) of the substrate samples was measured based on weight loss after the samples were dried at 101–105 °C to a constant weight (GB 5009.3-2016, China Standard [36]). The ratio of water to sample for pH determination was 10:1 (*v*/*w*). The beaker was shaken for 10 min and then left undisturbed for a standard 30 min before the pH value was measured with a pH meter (DB12T 729-2017, China Standard [37]). Organic carbon (OC) was measured via the potassium dichromate volumetric method and the external heating method (NY/T 1121.6-2006, China Standard [38]). Microbial biomass carbon (MBC), microbial biomass nitrogen (MBN), and microbial biomass phosphorus (MBP) were extracted via chloroform fumigation. Fumigating with chloroform at 25 °C for 24 h, the sample was extracted using a 0.5 M K_2_SO_4_ solution. After extraction, the MBC and MBN were determined via a carbon and nitrogen analyzer, and the MBP was determined via molybdenum-antimony resistance colorimetry. The total nitrogen (TN) content was determined via sulfuric acid-hydrogen peroxide digestion and the microKjeldahl nitrogen determination method (NY/T 2017-2011, China Standard [39]). The alkali-hydrolyzed nitrogen (AHN) content was determined via the alkalolytic diffusion method. Ammonium nitrogen (NH_4_^+^-N) and nitrate nitrogen (NO_3_-N) were extracted with a KCl solution and then analyzed by indophenol blue colorimetry and dual wavelength colorimetry, respectively. The total phosphorus (TP) content was determined via molybdenum-antimony resistance colorimetry (NY/T 2017-2011, China Standard). The content of quickly available phosphorus (QAP) was determined via the molybdenum-antimony resistance colorimetric method after hydrochloric acid extraction with sodium bicarbonate/sodium fluoride (NY/T 1121.7-2014, China Standard [40]).

### 2.3. DNA Extraction, PCR Amplification, and High-Throughput Sequencing

Upon arrival at the laboratory, each sample was immediately preserved at −80 °C. DNA extraction was completed with a DNA extraction kit according to the manufacturer’s instructions.

Library construction and sequencing: following the extraction of total sample DNA, the V3 and V4 hypervariable region of the 16S rRNA gene were amplified using the specific primers 341F (5′–ACTCCTACGGGAGGCAGCAG–3′) and 806R (5′–GGACTACHVGGGTWTCTAAT–3′) to investigate bacterial community composition and diversity, followed by the attachment of sequencing adapters and conserved regions to primer ends for PCR amplification. After product purification, quantification, and normalization, a sequencing library was generated for initial quality control, with the sequences that passed quality checks subjected to high-throughput sequencing via an Illumina NovaSeq 6000 platform. The resulting raw image data files were later transformed into raw sequencing reads through base calling. The findings were subsequently preserved in the FASTQ format (abbreviated as fq), which contained the sequence data as well as the respective quality data.

### 2.4. Data Processing and Analysis

Data preprocessing involved quality filtering and DADA2 denoising. First, the raw reads acquired were employed for filtering based on sequencing quality via Trimmomatic v0.33. Subsequently, Cutadapt 1.9.1 was used to identify and remove primer sequences, leading to clean reads. Second, we utilized the DADA2 approach within QIIME2 2020.6 for denoising and merging paired-end reads, together with chimeric sequence removal, ultimately yielding effective nonchimeric data. The culture materials were evaluated based on parameters such as microbial composition, diversity, and richness. When analyzing microbial diversity, we assigned samples to the smallest measurement unit to compare their differences. Subsequently, alpha and beta diversities were calculated, and the vegan R package was utilized for nonmetric multidimensional scaling to visualize and assess the distance matrix. The information analysis included feature classification (ASV level), diversity, differential, and correlation analyses (refer to the analysis results for details).

### 2.5. Statistical Analysis

All data for this study are presented as the means of 3 replicate measurements. Statistical analysis was performed via SAS software (version 9.1, SAS Institute Inc., Cary, NC, USA) via the general linear model (GLM) procedure. The least significant difference (LSD) at a 0.05 probability level was used to detect differences between treatments. Spearman correlation analysis was performed to analyze the relationships between the physicochemical properties and the different bacterial taxa via R language software (version 4.3.1), and a heatmap was drawn.

## 3. Results

### 3.1. Physicochemical Changes in the Substrate During V. volvacea Fruiting Body Development

**Figure 1:**
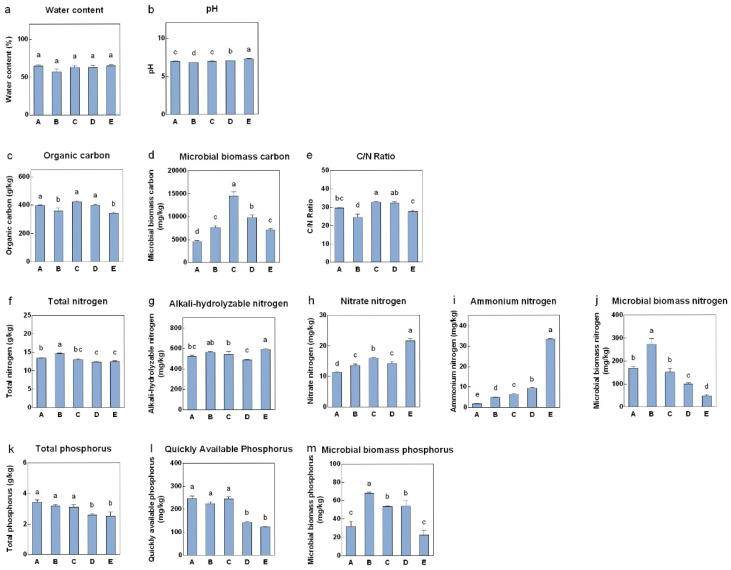
Physicochemical properties and nutrient contents of the substrate at different stages. (**a**) Water content; (**b**) pH; (**c**) organic carbon; (**d**) microbial biomass carbon; (**e**) C/N ratio; (**f**) total nitrogen; (**g**) alkali-hydrolyzed nitrogen; (**h**) nitrate nitrogen; (**i**) ammonium nitrogen; (**j**) microbial biomass nitrogen; (**k**) total phosphorus; (**l**) quickly available phosphorus; (**m**) microbial biomass phosphorus. A, B, C, D, and E represent the needle stage, the button stage, the egg stage, the elongation stage, and the maturation stage, respectively. Different letters indicate statistically significant differences.

During the different stages of fruiting body development, stages A, B, C, D, and E were 9, 12, 14, 16, and 18 days after sowing, respectively. There was no significant change in the WC of the substrate, whereas the pH initially decreased but then increased, remaining within the optimal range for the growth of *V. volvacea* (Figure 1a,b). OC, MBC, and the C/N ratio all peaked during stage C, with MBC showing a particularly significant increasing trend and then a decreasing trend throughout the fruiting body development stages (Figure 1c–e). This finding indicates that the absorption and utilization of carbon by the fruiting bodies increased from stages C to D. The TN content in the substrate also initially increased and then decreased, reaching the highest level during stage B, after which nitrogen was gradually absorbed and utilized by the fruiting bodies (Figure 1f). The trend in MBN was similar to that in TN, but the differences among the various stages were more pronounced (Figure 1j). In contrast, the AHN, NH_4_^+^-N, and NO_3_-N contents tended to increase during fruiting body development and were significantly greater during stage E than during the previous four stages (Figure 1g–i). The trends in TP and QAP were similar, remaining stable during stages A, B, and C and then significantly decreasing during stages D and E. These findings suggest that the absorption and utilization of phosphorus by fruiting bodies occurred mainly in stages C to D (Figure 1k,l). The content of MBP in stages B, C, and D was significantly greater than that in stages A and E (Figure 1m).

### 3.2. Microbial Community Diversity and Evolution

**Figure 2:**
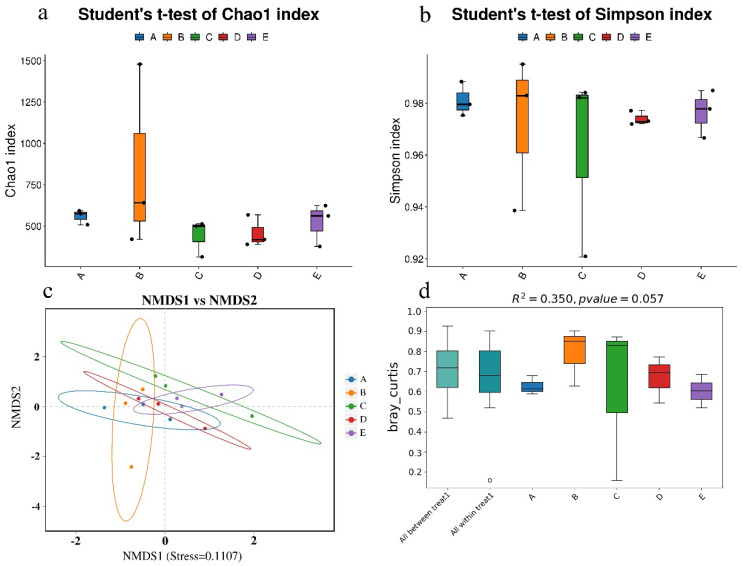
Alpha diversity and beta diversity of the substrate at different stages. (**a**) Alpha diversity based on the Chao1 index; (**b**) alpha diversity based on the Simpson index; (**c**) nonmetric multidimensional scaling analysis for the bacterial communities based on the Bray–Curtis similarity index; (**d**) PERMANOVA for the bacterial communities based on the Bray–Curtis similarity index. A, B, C, D, and E represent the needle stage, the button stage, the egg stage, the elongation stage, and the maturation stage, respectively.

To identify the genetic information of the microbial communities present during the development of *V. volvacea* fruiting bodies, 16S sequencing analysis was conducted on the substrate at five different stages. Alpha diversity reflects the richness and diversity of the microbial communities in samples. The box plots of the Chao1 index (Figure 2a) and Simpson index (Figure 2b) indicate that there were no significant differences in the richness or diversity of the bacterial communities across the five stages (*p* > 0.05). In this study, the Good’s coverage for all samples was above 99%, suggesting that all bacterial sequences in the samples were detected, accurately reflecting the actual composition of bacteria in the substrate.

Based on the substrates collected from the different stages, nonmetric multidimensional scaling (NMDS) ordination was performed (Figure 2c), and PERMANOVA was used (Figure 2d) to reveal dynamic changes in the microbial community composition. The results revealed that the identified taxa divided the substrate samples into five distinct groups, with group C and group D being completely separated, whereas the samples from the other groups overlapped, indicating differences in the bacterial community structure of the substrate at various stages of fruiting body development.

**Figure 3:**
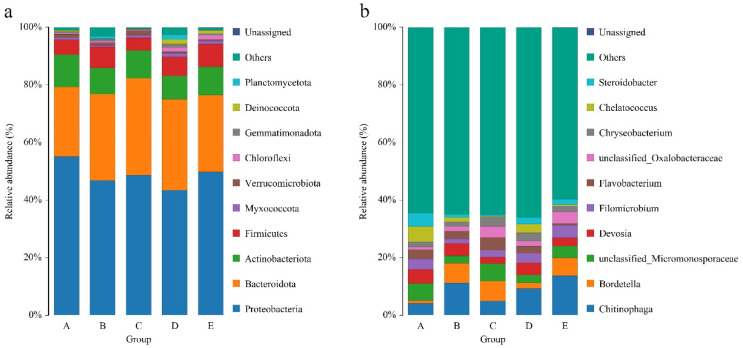
Taxonomic structure of the substrate bacterial community at the phylum (**a**) and genus levels (**b**). A, B, C, D, and E represent the needle stage, the button stage, the egg stage, the elongation stage, and the maturation stage, respectively.

Further differences in the bacterial community structure were detected at the phylum (Figure 3a) and genus (Figure 3b) levels. At the phylum level, the dominant phyla across the 5 stages were relatively consistent, with Proteobacteria consistently being the most abundant (relative abundance 43.40–55.07%). The relative abundances of Bacteroides, Actinobacteria, and Firmicutes followed, with relative abundances of 24.61–33.64%, 8.11–11.22%, and 4.33–7.89%, respectively. Among these, the trends for Proteobacteria and Actinobacteria were similar, with their relative abundances peaking in stage A and reaching their lowest values in stage D. In contrast, the abundances of Bacteroidota and Firmicutes peaked in stages C and E, respectively.

Unlike at the phylum level, different dominant genera were observed at various stages of fruiting body development, reflecting a more complex evolution of the community. *Chitinophaga* was the dominant genus in stages B, D, and E, with its relative abundance showing an increasing trend with fruiting body development, increasing from 4.19% in stage A to 13.67% in stage E. The dominant genus in stage A was unclassified_Micromonosporaceae (with a relative abundance of 5.95%), and its relative abundance peaked in stage C (6.00%). Similarly, the relative abundance of *Bordetella* (6.97%), which was the dominant genus in that stage, was the highest in stage C, and its relative abundance initially increased but then decreased throughout the fruiting body development process. This result is consistent with that of the beta diversity analysis, which revealed that the bacterial community structure in stage C significantly differed from that in the other stages.

### 3.3. Differences in Community Composition

**Figure 4:**
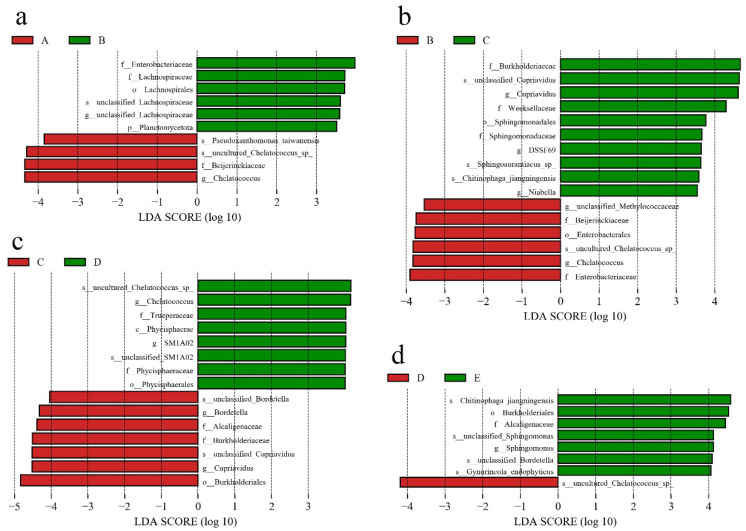
Taxonomic differences among substrate bacteria at different stages were determined via the linear discriminant analysis (LDA) effect size (LEfSe) method, and the LDA threshold was 3.0. (**a**) stage A vs. stage B; (**b**) stage B vs. stage C; (**c**) stage C vs. stage D; and (**d**) stage D vs. stage E. The X-axis represents the LDA score, and the greater the LDA score is, the greater the contribution of the taxon to differences between groups. The Y-axis lists different taxa, from genus to phylum, including unclassified taxa (such as unclassified taxa). A, B, C, D, and E represent the needle stage, the button stage, the egg stage, the elongation stage, and the maturation stage, respectively.

To identify bacterial taxa with significant differences in abundance between different stages of *V. volvacea* fruiting body development, a biomarker analysis based on the linear discriminant analysis (LDA), the effect size (LEfSe) method, was employed (Figure 4). There were 10, 16, 15, and 8 bacterial taxa showing significant differences between stages A and B, B and C, C and D, and D and E, respectively (the LDA threshold was 3.0). This indicated that the community compositional differences were greatest between stages B and C, followed by those in stages C and D, whereas the smallest differences were observed between stages D and E. In the comparison between stages A and B, taxa such as s-*uncultured*_*Chelatococcus*_sp., f-Beijerinckiaceae, and g-*Chelatococcus* were significantly enriched in stage A, whereas f-Enterobacteriaceae were significantly enriched in stage B. These bacterial taxa were significantly enriched in stage B according to the comparison between stages B and C. f-Burkholderiaceae, as well as s-*unclassified*_*Cupriavidus* and g-*Cupriavidus* belonging to this family, were enriched in group C in both the B vs. C and C vs. D groups, indicating that microbes in this family significantly contributed to differences in the microbial community among various stages of fruiting body development. In the stage D vs. E comparison group, taxa such as s-*Chitinophaga*_*jiangningensis*, f-Alcaligenaceae, and s-*unclassified*_*Bordetella* were significantly enriched in stage E, whereas only one species (s-*uncultured*_*Chelatococcus*_sp.) Interestingly, this species was also enriched in stages A, B, and D according to the other comparison groups, indicating its significant contribution to the differences in microbial community structure during the fruiting body development process.

### 3.4. Correlations Between Microorganisms and the Nutrient Profile of the Substrate

**Figure 5:**
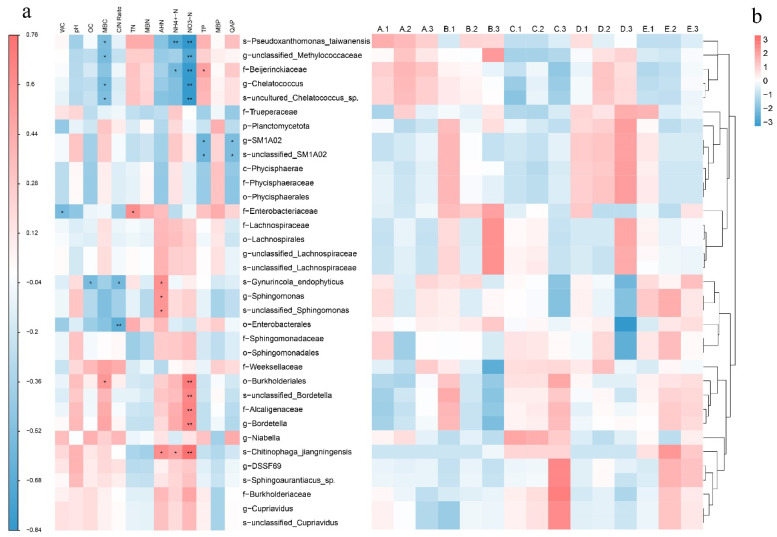
Heatmap of the relative abundance of different bacterial taxa (**a**) and correlation analysis between nutritional components and different bacterial taxa (**b**) in the substrate at different stages. Red and blue indicate positive and negative correlations, respectively. The intensity of the color represents the strength of the correlation, with darker shades signifying stronger associations. The analysis was conducted via Spearman’s correlation coefficient, and statistical significance is indicated by asterisks (* for *p* < 0.05, ** for *p* < 0.01). A, B, C, D, and E represent the needle stage, the button stage, the egg stage, the elongation stage, and the maturation stage, respectively, and 1, 2 and 3 represent the three repetitions for each stage.

After the bacterial taxa with significantly different relative abundances in the above comparison groups were selected, Spearman’s correlation analysis was conducted between these bacterial taxa and the nutritional components of the substrate. The results (Figure 5a) revealed that 10 bacterial taxa were significantly correlated with NO_3_-N, with 5 showing positive correlations and 5 showing negative correlations. This suggests that the microbial community structure had the greatest interaction with NO_3_-N. Additionally, five, four, three, three, two, and two bacterial taxa were significantly correlated with MBC, AHN, TP, NH_4_^+^-N, the C/N ratio, and QAP, respectively, and only one bacterial taxon was significantly correlated with OC, TN, and WC. No bacterial taxa were significantly correlated with pH, MBN, or MBP. Notably, all bacterial taxa significantly correlated with MBC were also significantly correlated with NO_3_-N. Among them, s-*Pseudoxanthomonas*_*taiwanensis*, g-unclassified-Methylococcaceae, g-*Chelatococcus*, and s-*uncultured*-*Chelatococcus*_sp. These bacteria were negatively correlated with MBC and NO_3_-N, and their abundance was relatively high in stage A but gradually decreased thereafter, reaching lower levels by stage E. In contrast, o-Burkholderiales was positively correlated with MBC and NO_3_-N, and its abundance showed the opposite trend, increasing with increasing cultivation stage (Figure 5b). Additionally, s-*Pseudoxanthomonas*_*taiwanensis*, f-Beijerinckiaceae, s-*Gynurincola*_*endophyticus*, and s-*Chitinophaga*_*jiangningensis* were significantly correlated with three nutrient indicators, suggesting that these bacterial taxa may play important roles in changes in the nutritional profile of the substrate. Overall, compared with carbon and phosphorus, nitrogen had a more pronounced interaction with the bacterial community structure.

## 4. Discussion

Researchers have previously suggested that *V. volvacea* bed cultivation outperforms cultivation in plastic bags and bottles [41]. The primary cultivation approaches for *V. volvacea* include indoor and greenhouse cultivation, followed by primary and secondary fermentation for 20–23 days of cultivation [42]. Owing to the short growth cycle of *V. volvacea*, its nutritional requirements differ from those of other edible fungi, and the nutrient content of the substrate is crucial for the growth and development of *V. volvacea*. Previous studies have focused mainly on the absorption and utilization of nutrients during the cultivation process of *V. volvacea*. In fact, during the development of *V. volvacea*, the demand for nutrients is greater in the fruiting body development stage than in the mycelial growth stage. Microorganisms coexist in the substrate and in the cultivation environment, and beneficial microorganisms facilitate macromolecular transformation and degradation or bioenergy utilization while suppressing the proliferation of diseases, bacteria, and insects [43]. These microorganisms interact with edible fungi, promoting the absorption and utilization of nutrients and significantly driving mycelial development and fruiting body formation [30].

Studies reporting the use of straw, mushroom residue, and waste cotton as primary cultivation materials for *V. volvacea* have noted variations in the bacterial community structure and the dominant microbial populations across different growth stages, with significant differences in microbial community structure and dominant populations observed depending on the cultivation material used [44,45]. Moreover, the microbial community in the cultivation environment gradually becomes enriched and diversifies over time [46]. In our study, we determined that the microbial community structure in the substrate changed during the development of the *V. volvacea* fruiting body, with these changes being more pronounced at the genus level. In agreement with studies on *Agaricus bisporus*, we found that the predominant bacterial phylum identified in all stages was Proteobacteria, followed by Bacteroidota [47,48,49]. However, there were different dominant genera at different stages of fruiting body development. The relative abundance of *Chitinophaga* increased with the development of fruiting bodies. Additionally, through NMDS analysis, we found that the bacterial community structure in Stage C significantly differed from that in the other four stages. The abundances of both Bacteroides and unclassified_Micromonosporaceae were highest in stage C. In research by Zheng, the abundance of Bacteroides, which is one of the dominant bacterial groups in surface soil, was significantly positively correlated with the degree of carbon metabolism [50]. Some genera of Micromonosporaceae have also been reported to participate in the carbon cycle by decomposing cellulose, hemicellulose, and other substances to provide energy for microorganisms and plants [51,52]. This is of vital importance for the growth of the *V. volvacea*, which is a type of straw-decay mushroom. An analysis of the nutrient composition of the substrate revealed that indicators related to carbon, such as OC, MBC, and the C/N ratio, all peaked in stage C, indicating that during stages A-B, the substrate accumulated a relatively high amount of C due to microbial activity, providing energy for the rapid growth of the fruiting bodies. As the fruiting body development has reached the egg stage, elongation stage, and maturity stage, the fruiting bodies significantly increased in size, consuming large amounts of energy. These results indicate that during the harvest period, the egg stage is the most important period in production. Notably, the trend in the MBC was the most evident. MBC represents the total amount of carbon contained within the microbial biomass of the substrate and is an important indicator for measuring soil microbial biomass, which is consistent with the results from NMDS analysis.

In addition to carbon, the contents of nitrogen and phosphorus in the substrate decreased due to the absorption and utilization of nutrients during the fruiting body development process. Moreover, the contents of readily absorbable forms of nitrogen, such as AHN, NH_4_^+^-N, and NO_3_-N, tended to increase, indicating that a greater amount of organic nitrogen decomposed into inorganic forms, increasing the nitrogen utilization rate. Correlation analysis between nutrient contents and microbial differences revealed that the bacterial community structure interacted most strongly with NO_3_-N, and the NO_3_-N content was significantly related to 10 bacterial taxa, such as Beijerinckiaceae and Burkholderiales. Warren noted that Beijerinckiaceae can mediate nitrogen fixation in the soil surface layer, providing plants with nitrate and other more readily absorbable forms of nitrogen [53], whereas Burkholderiales and Alcaligenaceae, as NO_3_-reducing microorganisms, have been reported to play important roles in the denitrification process in paddy field soil [54,55], which had the highest relative abundance at stage C in this study. Bacteria of the genus *Chitinophaga* have the ability to decompose chitin. Chitin is a polysaccharide found in fungal cell walls, and these bacteria decompose chitin by secreting chitinases, converting chitin into soluble sugars for other microorganisms and plants to utilize; this also contributes to the cycling of carbon, nitrogen, phosphorus, and other nutritional elements in the soil, thereby promoting the availability of plant nutrients. *Chitinophaga jiangningensis* has been reported to produce organic acids, releasing mineral elements such as Fe, Al, and Si and providing a variety of nutrients for plant growth [56]. In this study, *Chitinophaga jiangningensis* was also significantly related to AHN, NH_4_^+^-N, and NO_3_-N. These microorganisms work together to accelerate the nitrogen cycle in the substrate, maintaining the balance of nitrogen. Interestingly, among these bacterial taxa, five were significantly related to both MBC and NO_3_-N, and their relative abundance also clearly tended to increase or decrease with the development of the fruiting body. *Pseudoxanthomonas taiwanensis* can reduce nitrite to produce N_2_O, which is also an important part of the nitrogen cycle [57]. In addition, lignocellulose is a key natural polymer compound found in plant cell walls and represents a major structural component of plants. Lignocellulose has various key effects on plant development, environmental adaptation, and structural support [58]. Lignocellulose may promote matrix decay, assisting with nutrient adsorption in plants. Therefore, lignocellulose decomposition is necessary for fast and large-scale substrate production [59,60]. According to Wang’s study, *Pseudoxanthomonas taiwanensis* CB-226 has a strong lignocellulose degradation capacity and can decompose filter paper, cotton, rice straw, etc. [61]. In this study, the relative abundance of *Pseudoxanthomonas taiwanensis* increased with cultivation stage and was significantly related to NH_4_^+^-N, NO_3_-N, and MBC, which are important factors affecting the bacterial structure and nutritional profile of the substrate.

Although some studies have emphasized that nutrients are important for fungal development, this study confirmed these findings via omics technology, shedding more light on the relationships between fruiting body formation and the nutritional profile of the substrate. Through bioinformatics analysis, microorganisms that are important for *V. volvacea* growth and development were identified, laying a foundation for subsequent studies to identify microbial factors that regulate the yield of *V. volvacea*. Additional research should involve biochemical and physiological experiments to verify whether such microorganisms directly affect *V. volvacea* growth. These microorganisms are likely to positively affect production, indicating that increasing the production of *V. volvacea* through artificial increases in beneficial microbial populations and reductions in harmful populations is possible.

## 5. Conclusions

In this study, we investigated the nutritional profile and microbial community structure of the substrate during different stages of *V. volvacea* fruiting body development. Through correlation analysis between nutrient contents and microbial differences, we found that differential microbial taxa with functions such as nitrogen fixation, denitrification, and lignocellulose decomposition were significantly related to carbon- and nitrogen-related indicators such as NO_3_-N, MBC, and AHN. These microorganisms play a crucial role in the variation in the nutritional profile of the substrate, which caters to the nutrient requirements at different stages of fruiting body development. This study provides a theoretical basis for promoting the absorption and utilization of nutrients by *V. volvacea* by changing the structure of the substrate microbial community.

## Data Availability

The 16S rRNA V3-V4 amplicon sequencing data are available in the NCBI Short Read Archive (SRA) repository under accession number: PRJNA1260970.

## Abbreviations

WC	Water Content
OC	Organic Carbon
MBC	Microbial Biomass Carbon
MBN	Microbial Biomass Nitrogen
MBP	Microbial Biomass Phosphorus
TN	Total Nitrogen
AHN	Alkali-Hydrolyzed Nitrogen
NH^4+^-N	Ammonium Nitrogen
NO_3_-N	Nitrate Nitrogen
TP	Total Phosphorus
QAP	Quickly Available Phosphorus
DNA	Deoxyribonucleic Acid
PCR	Polymerase Chain Reaction
ASVs	Amplicon Sequence Variants
GLM	General Linear Model
LSD	Least Significant Difference
NMDS	Nonmetric Multidimensional Scaling
PERMANOVA	Permutational Multivariate Analysis of Variance
LEfSe	Linear Discriminant Analysis Effect Size
LDA	Linear Discriminant Analysis
NCBI	National Center for Biotechnology Information
SRA	Sequence Read Archive
